# Microvascular errors of technique: a systematic review

**DOI:** 10.1007/s00701-026-06810-w

**Published:** 2026-03-08

**Authors:** Victor Esanu, Elisa Marziali, Oana Gaspar, Stefan Agoston, Teona Carciumaru, Alexandra Stoia, Claudia Paun, Horatiu A. Colosi, George Dindelegan, Clemens Dirven, Torstein R. Meling, Dalibor Vasilic, Victor Volovici

**Affiliations:** 1 Erasmus MC, Rotterdam, Netherlands; 2 Iuliu Hațieganu University of Medicine and Pharmacy, Cluj-Napoca, Romania; 3 Rigshospitalet, København, Denmark

**Keywords:** Microvascular anastomosis, Cerebrovascular bypass, Technique errors

## Abstract

**Introduction:**

Microvascular procedures demand exceptional precision and are prone to technical errors that compromise outcomes. Performance improves fastest when errors are identified, measured, and corrected early during training. Error-based learning has become an essential part of microsurgical training, highlighting the importance of identifying and learning from mistakes to improve performance. This review aimed to systematically search the literature on all microsurgical errors and categorize them by operative phases.

**Materials and methods:**

A structured literature search was conducted across Medline, Embase, and Web of Science databases, following PRISMA guidelines. Two reviewers independently screened records and extracted data in duplicate. Articles were included if they evaluated microvascular anastomoses with vessels less than 2 mm in diameter, and if microsurgical errors were detailed along with their impact on outcomes, in particular on anastomotic patency. Given the heterogeneity of the data, a SWiM-style (Synthesis Without Meta-analysis) narrative synthesis was used.

**Results:**

A total of 34 studies met the inclusion criteria. Errors were categorized as pre-operative, intra-operative, and post-operative. Intra-operative errors were the most frequently reported. Back-wall stitches, uneven lumens, and excessive suture tension were consistently associated with reduced patency. Several validated scoring tools (e.g., ALI, MARS10, OSATS) were identified as effective in quantifying errors and guiding feedback in training settings.

**Conclusion:**

Microvascular anastomosis errors span all phases of the microsurgical procedure and significantly affect anastomotic success. This review offers a structured taxonomy of errors and underscores the importance of error-based learning and assessment in microsurgical training. Standardized error classification may enhance training programs, accelerating the acquisition of microsurgical skills along the learning curve and improving clinical outcomes.

**Supplementary Information:**

The online version contains supplementary material available at 10.1007/s00701-026-06810-w.

## Introduction

Microsurgery remains one of the most challenging surgical techniques to master, requiring a steep learning curve along with exceptional dexterity [38]. As the range of microsurgical procedures expands, structured training approaches that emphasize error recognition linked to correction are increasingly important to accelerate skill acquisition while protecting outcomes [14, 18, 28].

Microsurgical training is delivered across living and non-living models. Most microsurgical courses worldwide train on living models, such as Brown Norway rats, which provide realistic tissue handling and immediate patency feedback but are constrained by cost and ethical concerns [3, 13, 42, 46]**.** In contrast, non-living models are accessible for early skill building, but cannot provide thrombosis feedback [43].

Mastering microsurgical technique demands rigorous training, which has led to the development of highly specialized and complex courses [12, 29]. Error-based learning has become an essential part of microsurgical training, highlighting the importance of identifying and learning from mistakes to improve performance [33]. As a result, a dedicated branch of microsurgical training has emerged, concentrating on the systematic identification of errors and their correlation with outcomes, such as anastomosis patency. To support this focus, various error assessment tools and scoring systems, including Anastomosis Lapse Index (ALI) [15, 16], the Global Rating Scale (GRS), and the Microsurgical Anastomosis Rating Scale (MARS10) [43] have been developed. These tools enable objective evaluation of microsurgical skill acquisition and provide a structured framework for tracking progress and identifying areas for improvement.

A summum of errors is at the heart of performance breakdowns in microsurgery, yet definitions, causes, and their links to outcomes are inconsistently reported. Defining and quantifying error types are essential steps for a faster, more efficient learning curve [44]. The aim of this review was to systematically collect and analyze existing literature on both microsurgical errors as well as their impact on anastomosis outcomes. By synthesizing these findings, we aim to support more targeted training and assessment in microsurgical education.

## Materials and methods

The methodology of the systematic review was predefined and outlined in a protocol, provided in Supplementary Appendix 1.

### Search strategy

The literature search was conducted with the assistance of a specialist librarian from Erasmus MC University Medical Center, Rotterdam. Three databases were searched (Medline, Embase, and Web of Science). A complex, tailored search strategy was employed, focusing on key terms such as "microsurgery," "errors," and "anastomosis" (Supplementary appendix 2).

All identified articles were uploaded to the Covidence systematic review software (Veritas Health Innovation, Melbourne, Australia. Available at www.covidence.org). Duplicated entries were removed, and the remaining studies were screened separately by two reviewers (EM, OG).

### Selection and eligibility criteria (PICOS)

The review included studies published in English which involved trainees or surgeons who performed microvascular anastomoses of vessels less than 2 mm in diameter. It included studies in simulation, animal, or clinical settings. Eligible studies evaluated training components of vascular microsurgery and reported end-product assessments focusing on microsurgical errors, with errors clearly described. Some studies also analyzed the relationship between errors and clinical outcomes, such as anastomotic patency, where possible. The outcomes considered included objective error analysis, patency or flow measures, and validated assessment tools such as ALI or MARS10. Eligible study designs comprised randomized controlled trials, non-randomized comparative studies, as well as observational and historical control studies.

Articles were excluded if they: 1) did not specifically investigate microvascular anastomoses, such as those primarily addressing microsurgical applications in otolaryngology, maxillofacial surgery, or ophthalmology; 2) they evaluated microsurgical techniques without assessing or describing specific end-product errors or mistakes; 3) they lacked a clear definition and detailed description of the microsurgical errors and did not analyze their relationship with outcomes in any way, particularly anastomotic patency; or 4) were not published in the English language.

The study selection process followed the Preferred Reporting Items for Systematic Reviews and Meta-Analyses (PRISMA) guidelines. Titles and abstracts were screened by two independent reviewers (EM, OG) to identify studies that met the inclusion criteria. Searches ran from inception to 27 March 2025. Reference lists were also screened. Conflicts were resolved by a third reviewer (VV), blinded to the prior assessments. After consensus was achieved among reviewers, the selected articles were screened for full-text. Besides the articles generated by the search, additional articles were backtracked from the reference lists of included articles and imported into Covidence for screening.

### Data extraction and analysis

We defined errors as observable end-product deviations (e.g., back-wall stitch), and their causes as contributory factors, such as human, technical, environmental, or anatomic conditions that increase error likelihood (e.g., distraction, inappropriate use of the operative field).

Data was extracted from the articles by the two reviewers independently, in duplicate (EM, VE), using a standardized form. Extracted information included types and classifications of microsurgical errors, scoring systems or tools used, and the reported outcomes, such as anastomosis patency, thrombosis rates, or training performance. Discrepancies were resolved by consensus.

Given the heterogeneity in study designs and outcome measures, a prespecified SWiM (Synthesis Without Meta-analysis) approach was adopted [2]. Data was synthesised narratively, with errors being grouped according to pre-, intra-, and postoperative phases. The intra-operative phase was further subdivided into dissection, needle passage, and suture domains. The quality assessment (risk of bias) and detailed study characteristics are provided in Supplementary Appendices 3 and 4.

## Results

**Fig. 1:**
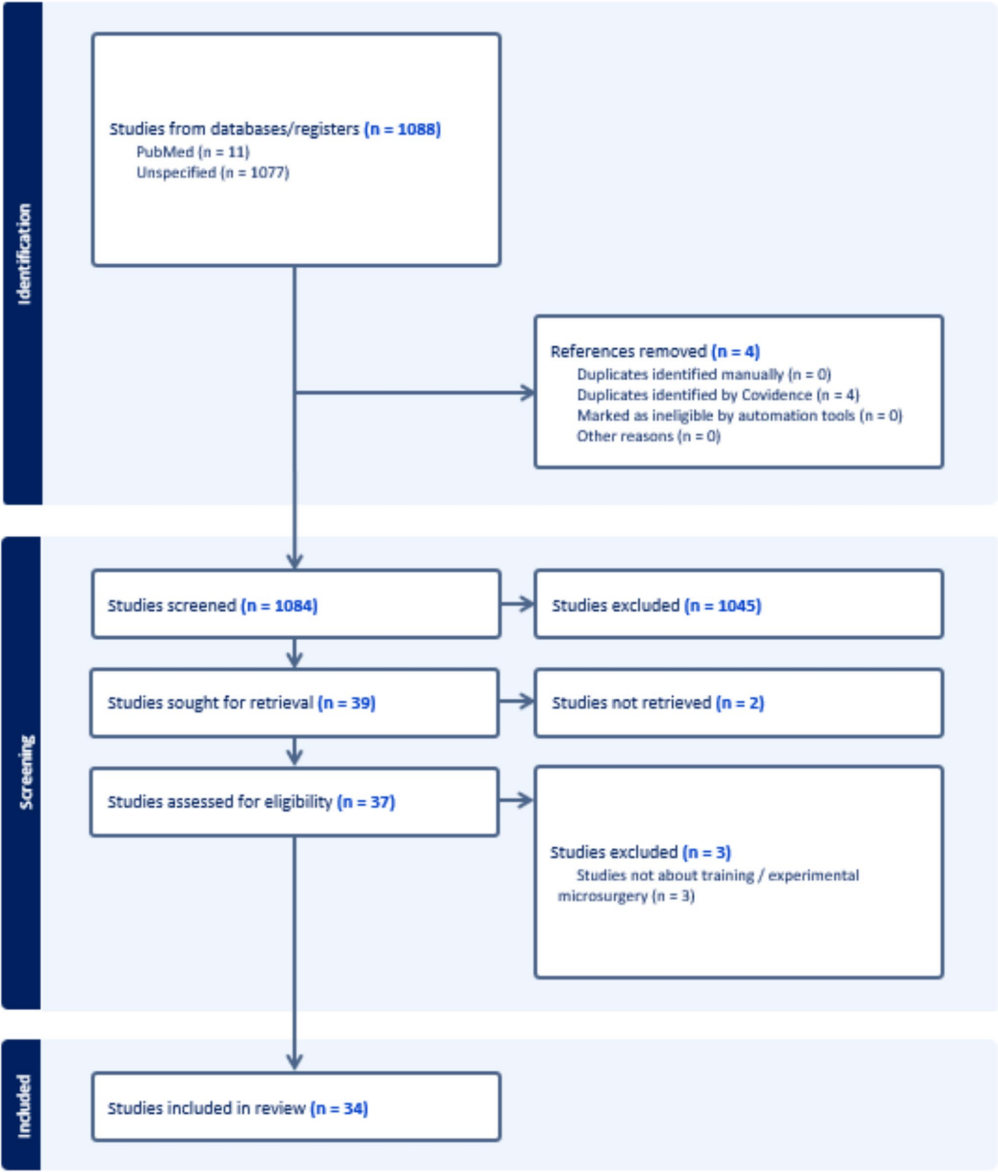
PRISMA flowchart of the reference selection process

The initial search of the MEDLINE, Embase, and Web of Science databases retrieved 1,088 results. Four duplicate references were identified by Covidence and removed. After title and abstract screening of the 1,084 articles, 39 were selected for full-text assessment. Of these, two articles were unavailable, and three were excluded for not meeting the inclusion criteria. In total, 34 articles were included in this review (Fig. 1). Errors were categorized according to the operative phase: preoperative, intraoperative, and postoperative.

### Pre-operative phase

Ten studies reported errors related to the pre-operative phase. These included both setup-related issues (Table 1) and external factors that could influence microsurgical performance. Setup-related errors involved incorrect instrument selection, poor microscope calibration, and inadequate preparation of the operative field. Several studies described technical handling problems caused by suboptimal setup of the operative field, such as repeated regrasping or failure to properly remove instruments from the field of view.

**Table 1:** Classification of errors occurring during the pre-operative phase

Pre-operative Errors	
Errors related to tool selection	Errors related to the calibration of the microscope
Empty grasp [7, 9, 41]	Inappropriate operative field [7, 9, 26, 41]
Regrasping [39]	Loss of focus [7, 9, 26, 41]
Improper selection of the needle and suture size to the vessel size [34]	Loss of central view [7, 9, 26, 41]
Inappropriate use of instruments [39]	Inappropriate magnification [7, 9, 26, 41]
Repeated attempts/awkward moves [39]	

External influences included distractions in the operating environment and insufficient mental preparedness before the procedure. These findings were observed consistently across both simulation-based and in vivo models.

### Intra-operative phase

The review identified 33 articles addressing intra-operative errors (Table 2). For better clarity, the intra-operative errors were further classified into three categories: 1) errors during vessel dissection and preparation, 2) errors related to needle passage through tissues—including improper needle entry and exit, and 3) errors associated with suture handling. Errors in vessel dissection and preparation were further classified as inadequate exposure of the surgical site, errors in vessel preparation, and incorrect vessel positioning.

**Table 2:** Overview of intra-operative errors

Intra-operative Errors							
Errors in vessel dissection and preparation phases							
Inadequate or insufficient exposure of the surgical site		**Errors in vessel preparation**			Incorrect vessel positioning		
Limitation of clamping space [25]		Inappropriate vessel setup [7, 9, 41]			Vessel clamp reapplication [7, 9, 26, 40, 41]		
Awkward surgical field [25]		Vessel desiccation [7, 9, 41]			Insufficient approximation [44]		
Excessively wet field [26]		Insufficient dissection [7, 9, 26, 41]			Inadequate vessel tension [10, 23]		
		Inadequate trimming of the adventitia [32]			Torsion [17, 19, 24, 25, 45]		
		Improper dilation of the vessel [32]			Twisting of the pedicle [19]		
Errors during needle passage through tissue							
Catching more than one wall	**Inappropriate bite size**	**Poor needle handling**	**Vessel trauma**	**Failure to pass through all layers**		**Suture spacing**	Suture orientation
Backwall suture [1, 4, 5, 7–9, 15, 19, 21, 24, 26, 27, 30–32, 34, 35, 37, 41, 43, 44]	Unequal bites causing tissue enfoldment [1, 4, 5, 7, 9, 11, 15, 23, 36, 40, 41, 43, 47]	Needle suture tear [1, 4, 5, 7, 9, 15, 26, 32, 35, 36, 39, 41, 44]	Wrong grasp causing tissue damage [7, 9, 40, 41]	Partial thickness suture [1, 4, 5, 7–9, 15, 23, 26, 27, 32, 35, 36, 44]		Suture gaps [1, 4, 5, 15, 36]	Oblique sutures [1, 4, 5, 8, 15, 27, 35, 36]
Sidewall suture [1, 4, 5, 8, 15, 26, 27, 35–37]		Failing to pull the needle out of the field [39]	Traumatic grasping of vessel margins [25, 34, 39]	Adventitia only suture [1, 36]		Over-suturing [1, 7, 9, 26, 36, 41]	Disruption of anastomosis line [1, 4, 5, 15, 21, 36, 39]
			Intimal damage [23, 45]				Asymmetric spaces between sutures [7–9, 11, 22, 23, 25, 26, 32, 35, 39, 43]
Errors during suture handling (thread unwinding and knot-tying maneuvers)							
Improper suture tension	**Wrong number or direction of loops**	**Residual material in vessel**	**Too rapid thread unwinding**	**Suture cutting errors**		Incorrect handling of the suture	
Tight suture tear (Cheese wire tear) [1, 5, 7, 9, 15, 26, 34–36, 40, 41, 44]	No square knot (inadequate technique) [26, 32, 39, 43]	Suture material in lumen [1, 4, 5, 7–9, 11, 15, 19, 35, 36, 39, 41, 43]	Suture pulled through that needs to be redone [7, 9, 26, 41]	Suture cut through [7, 9, 41]		Broken suture [7, 9, 26, 32, 41]	
Loose sutures [7, 9, 32, 39, 40, 43]				Suture tails cut too short/long [26, 32, 39, 43]		Knotted suture [7, 9, 32, 41]	
Tissue strangulation [4, 5, 7, 9, 15, 19, 23, 32, 35, 43]						Inadequate loop length [7, 9, 32, 41]	
						Inadequate pressure [26, 32]	
						Excessive suture traction [26, 32]	

Errors than can occur during needle passage through tissue were classified as back-wall/side-wall stitches, wide bite sizes, vessel wall tearing, sutures that do not pass through the full thickness of the vessel wall (partial thickness bites), unequal distances, disruption of the anastomosis line, oblique sutures, and large edge overlaps.

Errors in suture handling include improper tension, incorrect loop numbers affecting knot integrity, intraluminal thread residues causing thrombosis, fast thread unwinding leading to repositioning and extra needle holes prone to further bleeding after unclamping, accidental cutting of the knot or vessel, and mishandling that damages the needle or thread for subsequent sutures.

### Post-operative phase

Eight articles included in this systematic review reported post-operative errors related to microsurgical anastomoses. The studies identified various issues occurring after the completion of the procedure, primarily concerning patency assessment and early post-anastomotic complications. An overview of the post-operative errors extracted from these articles is presented in Table 3.

**Table 3:** Classification of post-operative errors

Post-operative Errors		
Vessel trauma during patency tests	Premature declamping of the artery	Vascular obstruction
Crushing patency tests [7, 9, 41]	Anastomotic leak [7, 9, 26, 35, 40, 41]	Impeded flow [7, 9, 41]
	Pooling of fluid [7, 9, 41]	Mural thrombus [25, 27]
		Completely obstructed anastomosis [43]

A meta-analysis could not be conducted due to a lack of data that can be pooled. Findings were presented as a narrative synthesis. Most studies provided high-credibility descriptive error reporting (ECC) and a subset of studies quantified error–outcome associations (EOAC). Full ratings can be found in Supplementary Appendix 3.

**Fig. 2:**
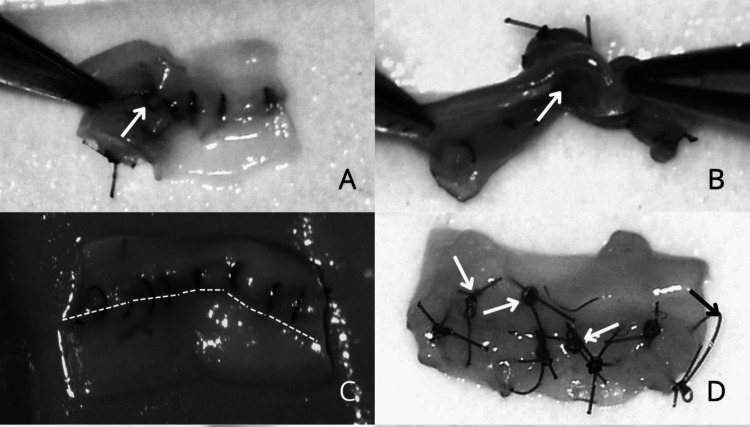

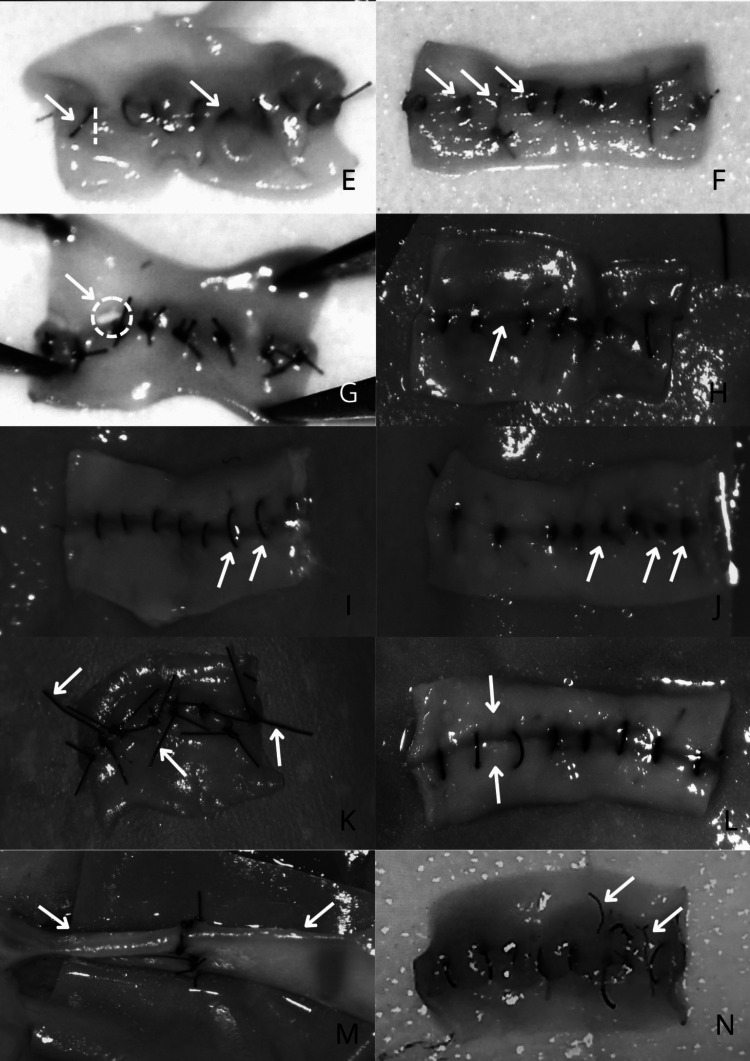
Measurable errors during end-product assessment. **A** Backwall stitch, **B** Sidewall stitch, **C** Disruption of anastomosis line, **D** Loose knots, **E** Oblique stitch, **F** Partial thickness bite, **G** “Cheesewire” tear, **H** Large distance between two knots, **I** Wide/large bite, **J** Excessive tightening, **K** Moustache sign, **L** Tissue overlap, **M** Vessel twist, **N** Thread in lumen

**Fig. 3:**
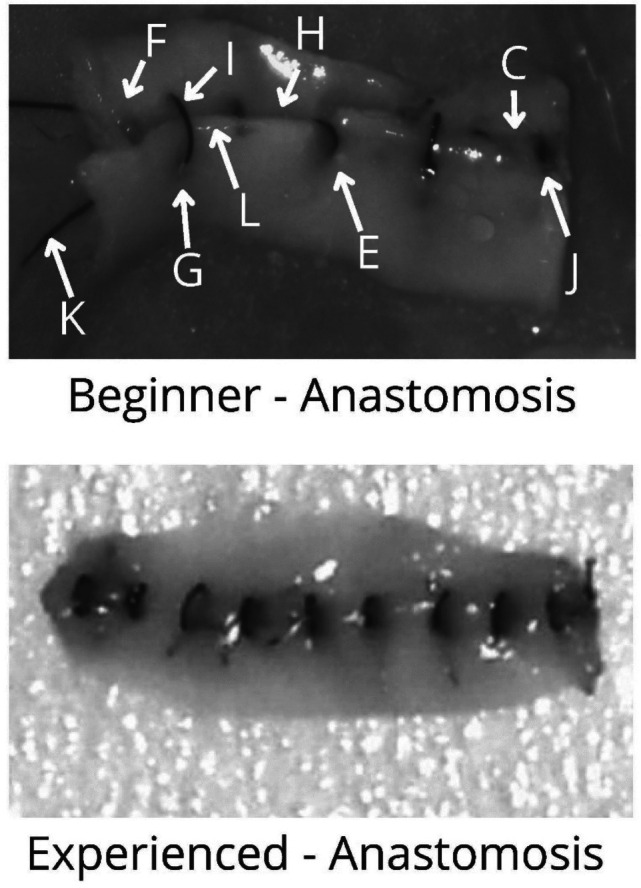
Beginner vs experienced end-product assessment of errors. C) Disruption of anastomosis line, E) Oblique stitch, F) Partial thickness bite, G) “Cheesewire” tear, H) Large distance between 2 knots, I) Wide/large bite, J) Excessive tightening, K) Moustache sign, L) Tissue overlap

To aid interpretation, we provided in Figs. 2 and 3 schematic illustrations of errors during end-product assessment, which visually summarize the most frequent and objectively measured microvascular errors identified during this review.

## Discussion

### Summary of results

This systematic review analyzed 34 studies addressing microsurgical errors across the pre-operative, intra-operative, and post-operative phases. Intra-operative errors were the most frequently described, mainly involving vessel dissection, needle handling, and suture management. Pre-operative errors were related to operative setup or cognitive distractions, while post-operative errors involved patency assessment and management decisions. This review highlights key areas for targeted training and quality improvement in microsurgical education.

### Pre-operative errors

Most pre-operative errors are caused by insufficient preparation before the intervention, meaning error prevention needs to start before the microscope is even in use. The majority of studies prioritize end-product assessment, often overlooking this critical component. Inadequate microscope calibration, such as improper magnification, loss of focus, or misaligned operative field can predispose to mistakes [7, 9, 26, 41]. Tool-related issues like regrasping and improper instrument use further highlight persistent challenges in technique and ergonomics [7, 9, 34, 39, 41], suggesting that careful setup and focused training may reduce error rates. Chan et al. [7] showed via the Structured Assessment of Microsurgery Skills (SAMS) methodology that microsurgical errors arise not only from technique but also from planning, operative flow, and visuo-spatial factors, highlighting the cognitive dimension of errors. Carr et al. [4] studied the effects of ORDI (operating room cognitive distractions) and concluded that operating under stress led to faster completion but lower accuracy, while Chadha et al. [6] demonstrated that mental rehearsal before operating reduced the number of errors. These findings underscore the importance of thorough preoperative assessment, as careful planning and equipment setup can reduce microsurgical errors. Future studies should explore structured preoperative strategies and their impact on error prevention.

### Intra-operative errors

Intraoperative procedures represent the stage most susceptible to errors, with the majority of mistakes occurring during real-time vessel manipulation and dissection. During vessel dissection and preparation, they appear largely related to vessel handling. Incorrect vessel positioning was most frequently reported, with issues such as repeated clamp reapplication [7, 23, 26, 39, 41], torsion [17, 19, 24, 25, 45], and excessive vessel tension [10, 23] highlighting the technical challenges and precision required in microsurgery. These observations suggest that targeted training in vessel manipulation could reduce common intraoperative mistakes.

Backwall catches, described in 21 articles, were the most frequent errors during needle passage, followed by irregular suture bites, needle-induced suture tears, partial-thickness bites, and tight suture tension. This further emphasizes that needle handling, suture integrity, and tension management are critical determinants of microsurgical success.

Intraoperative errors, consistently reported across multiple studies, are the most common and reliable indicators of microsurgical performance. Their frequency highlights the intraoperative phase as the critical period where technique directly affects anastomosis integrity. Focusing training on reducing these errors could therefore have a significant impact on improving microsurgical skills.

### Error assessment and scoring systems

Over time, several scoring systems have been introduced to assess microsurgical performance, with a particular focus on detecting technique errors. The ALI (Anastomosis Lapse Index), developed by Ghanem et al. [15], is among the most frequently used tools for end-product evaluation, likely due to its ability to capture common and clinically relevant error types. Paladino et al. [36] further applied the ALI score in the rat microvascular model, noting that the model may tolerate certain technical errors, which raises concerns about its sensitivity in accurately reflecting surgical precision. This observation supports the idea that training should prioritize error recognition and stitch accuracy rather than relying solely on patency as an endpoint.

Additionally, Pafitanis et al. [35] evaluated the Intimal Surface Suture Line Assessment (ISSLA Error List) in end-to-side anastomosis, categorizing errors by severity (high, medium, low) to provide structured guidance for correction.

Together, these tools reflect a growing shift toward systematic, error-based assessment. However, there remains a need to develop a comprehensive, large-scale scoring system that captures all potential errors throughout every phase of microsurgery, from preparation to final evaluation, in order to better objectify training. Such a standardized framework would not only support a more targeted skill development and improve clinical outcomes but also reduce the reliance on animal models by enabling more efficient learning algorithms for trainees entering microsurgical practice.

### Mechanical factors: Torsion and tension

Mechanical factors, especially torsion and tension, can determine microsurgical success. Across experimental models, severe torsion may reduce patency, but limited torsion (≤ 180°) often shows no clear early patency penalty, so any twist should still be actively identified and corrected intraoperatively [17, 19, 20, 24, 45]. In contrast, excessive anastomotic tension is more consistently associated with failure, with patency preserved only when less than 20 percent of the length of the vessel was resected [10]. These issues are most relevant in bypasses and free flaps, where small alignment or tension errors can compromise perfusion, making routine intraoperative checks essential.

### Technique comparisons and specific errors

Technique comparisons highlight how surgical approach influences precision and training efficiency. Kim et al. [21] found no significant differences between biangulation and triangulation in terms of patency, error rate, or suture quality, indicating that both are suitable for beginners. Catching more than one vessel wall remains one of the leading causes of occlusion. Nasir et al. [30] showed that a purposeful back-wall stitch increased thrombosis in femoral arteries. An interesting observation has to be mentioned, made by Pignatti et al. [37] who found no significant impact when a two-wall stitch was performed in veins, suggesting that vessel characteristics may modify the consequence of this error.

Advancements in technique, such as the Double Stitch Everting method [11], and the Needle Splint technique introduced by Pafitanis et al. [35], have demonstrated improved suture symmetry and eversion with reduced operative time. Another important technique aspect is tail-length control when knot-tying, which serves as an indicator of proficiency, economy, and workflow planning, that can be trained and assessed.

These findings emphasise the importance of refining surgical technique to minimise prevalent errors and optimise microsurgical training outcomes, highlighting the need to study multiple techniques to identify the most effective approaches for novice training.

### Post-operative errors

Postoperative errors often involve premature artery opening before adequate coagulation or blood flow is established. Fluid pooling was reported in three studies [7, 26, 41] and anastomotic leaks in six [7, 26, 27, 39, 41]. Chan et al. [7] using the SAMS methodology, classified judgment errors, including crushing patency tests, anastomotic leaks, fluid pooling, and impaired flow requiring reanastomosis. These errors remain relatively understudied despite their impact on surgical outcomes.

### General guidelines

On specific strategies that help improve anastomotic quality and patency rates, Nimmons et al. [32] have developed the Objective Structured Assessment of Technical Skills (OSATS) scoring system, the microvascular arterial anastomosis task, which is available in Table 4. It comprises a task-specific checklist and a Global Rating Scale. In their study, 20 surgeons with different skill levels in microsurgery performed end-to-end arterial anastomoses on a chicken thigh model. They concluded that certain tasks are associated with a higher quality of the anastomosis. Although OSATS is not a microsurgical error scale, it serves as a guideline for an error-free microsurgical anastomosis and stands as a reliable standard for evaluating and refining essential microsurgical skills.

**Table 4:** Microvascular objective structured assessment of technical skills (OSATS)—task specific score

Skill Evaluated	Correct/Incorrect
1. Loads needle in drive 1/2 to 2/3 from needle tip	
2. Needle does not wobble in driver	
3. Needle enters tissue perpendicularly	
4. Forceps handle vessel adventitia to provide counter traction	
5. Dilator is appropriately used	
6. Needle is pulled through tissue following its curve	
7. Suture is pulled out parallel to the tissue	
8. Suture tails are left at the correct length	
9. Appropriate depth tissue bite on each side	
10. Sutures are spaced appropriately	
11. Three or more square throws are tied	
12a. Efficient handling of suture while tying	
12b. Appropriate tail length before tying (not too long/short)	
13. Appropriate tension on suture while tying	
14. Tissue well-approximated but not strangulated	

### Limitations

This systematic review has several limitations. The microsurgical errors identified were highly heterogeneous, varying in characteristics, severity, and context, which made developing standardized rubrics challenging. Due to this heterogeneity, a non-validated risk of bias tool was used. While some underlying causes of microsurgical errors were addressed, not all could be encompassed. For instance, mural thrombus formation was classified as a postoperative mistake, though pre- or intraoperative factors like inadequate vessel preparation or poor suture technique could also contribute. The review mainly focused on end-product assessment, which may not fully capture all causal factors. Future research should correlate specific technical or procedural shortcomings with their resulting errors and systematically map these within the operative workflow to better inform prevention strategies and structured training programs.

## Conclusion

This systematic review identified a comprehensive classification for microsurgical errors reported in literature. By focusing on the operative steps, an objective categorization of the various errors encountered in studies was achieved. This approach allowed the creation of a structured system for understanding these mistakes and their implications. This article may serve as a valuable resource in consolidating the spectrum of microsurgical errors into a single reference, ultimately contributing to improving microsurgical training through error-based learning.

## Supplementary Information

Below is the link to the electronic supplementary material.

ESM 1 Supplementary Material 1 (DOCX 23.2 KB)

ESM 2 Supplementary Material 2 (DOCX 15.5 KB)

ESM 3 Supplementary Material 3 (XLSX 532 KB)

ESM 4 Supplementary Material 4 (XLSX 25.6 KB)

ESM 5 Supplementary Material 5 (PDF 171 KB)

## Data Availability

The extraction sheet, consensus ECC/EOAC ratings, PRISMA flow, and other materials are available at Zenodo link.

## Abbreviations

*ALI*: Anastomosis Lapse Index
*ECC*: Error Cataloguing Credibility
*EOAC*: Error–Outcome Association Credibility
*Erasmus MC*: Erasmus Medical Center
*GRS*: Global Rating Scale
*MARS10*: Microsurgical Anastomosis Rating Scale (10-item)
*ORDI*: Operating Room Cognitive Distractions
*OSATS*: Objective Structured Assessment of Technical Skills
*PRISMA*: Preferred Reporting Items for Systematic Reviews and Meta-Analyses
*SAMS*: Structured Assessment of Microsurgery Skills
*SWiM*: Synthesis Without Meta-analysis
