# Supplementary material for: Microvascular errors of technique: a systematic review
Source: Acta Neurochir (Wien). 2026 Mar 8;168(1):65. doi: 10.1007/s00701-026-06810-w (PMC12971797; doi:10.1007/s00701-026-06810-w)
Supplement: Supplementary file 1 — Supplementary Material 1 (DOCX 23.2 KB) [file 701_2026_6810_MOESM1_ESM.docx]

**Supplement S1. Protocol (PRISMA-P–aligned)**

**Title:** Microsurgical mistakes: A Systematic Review of Types of Errors and their causes
**Short title:** Protocol—Microvascular Anastomosis Errors
**Registration:** Not registered in PROSPERO (protocol developed a priori)
**Contributors:** [VE],[EM],[OG],[SA],[AS],[CP],[TC],[HAC],[GCD],[CD],[TRM],[DV],[VV]
**Support/funding:** None.
**Competing interests:** None declared.
**Contact:** Corresponding author: Victor Volovici, MD, PhD - v.volovici@erasmusmc.nl

**1) Rationale**

Efficient microsurgical training depends on making errors observable and measurable, identifying their common causes early, and using that knowledge to build error-oriented training programmes that accelerate the learning curve and improve error recognition and remediation.

**2) Objectives (PICOS framed)**

**Primary objective:** Identify and classify **error types** in microvascular anastomosis and summarise their **association with end-product outcomes** (e.g., patency/flow).
**Secondary objective:** Synthesize **contributory factors/causes** linked to these errors (technical, cognitive, environmental, equipment, anatomic/biomechanical) across pre-, intra-, and postoperative phases.

**3) Eligibility criteria (PICOS—operational)**

- **Population:** Human learners/clinicians (students, residents, surgeons) *and/or* models used to train/test microvascular anastomosis (in-vitro/synthetic, ex-vivo, animal).
- **Intervention/Exposure:** Microvascular anastomosis procedures/tasks (arterial or venous) with explicit **error definitions** (e.g., back-wall stitch, partial-thickness bite, excessive tension, torsion) and/or **contributory factors**.
- **Comparator:** Baseline, alternative technique/programme, or none.
- **Outcomes (pre-specified):**
  - **Primary:** Objective end-product outcomes (e.g., **ALI**, **MARS10**, leak/bleed, patency/flow tests at defined time points).
  - **Secondary:** Error frequency/severity; contributory factors; retention/follow-up
- **Study designs:** RCTs, non-randomised comparative, before–after, cross-sectional/observational in simulation, animal, or clinical settings.
- **Limits:** English language; no date limits.
- **Exclusions:** Editorials, narrative reviews, case reports without defined error taxonomy; studies not involving microvascular anastomosis.

**Construct definitions (used consistently):**

- **Error:** Observable deviation in process/product (e.g., back-wall stitch, uneven bite, knot insecurity).
- **Contributory factor (“cause”):** Conditions that increase error likelihood (technical, cognitive/attentional, environmental/distraction, equipment, anatomic/biomechanical).

**4) Information sources**

- **Databases:** MEDLINE (Ovid), Embase (Embase.com), Web of Science Core Collection.
- **Coverage:** From inception to **25^th^ March 2025** (final search date).
- **Other sources:** Reference lists of included studies and pertinent reviews. (No formal grey-literature or trial-registry searches planned due to expected low yield in this education/procedural domain.)

**5) Search strategy**

Database-specific strategies (controlled vocabulary and keywords for *microsurgery/microvascular*, *anastomosis*, *error/mistake/complication*, *patency*) are provided in **Supplement S2.** No date or study-design filters were applied beyond the language limit

**6) Study records**

**6.1 Data management**

Results were exported to a reference manager for de-duplication, then imported into **Covidence** for screening, selection tracking, and data extraction.

**6.2 Selection process**

Two reviewers (**[EM]**, **[OG]**) independently screened **titles/abstracts** and **full texts** against eligibility. Disagreements were resolved by **discussion** (pre-specified adjudicator: senior author **[VV]**). Reasons for full-text exclusion recorded and displayed in the **PRISMA 2020 flow diagram**.

**6.3 Data collection process**

Two reviewers (**[EM]**, **[VE]**) **extracted data in duplicate** using a piloted form, as seen from the text Tables. Discrepancies were resolved by discussion; consensus data were used for analysis.

**7) Data items (extraction fields)**

- **Study descriptors:** author, year, country, setting (simulation/animal/clinical), single- vs multi-centre, design.
- **Participants/model:** learner level (student/resident/surgeon), sample size; species/model (rat/chicken/synthetic); vessel type/diameter.
- **Error taxonomy:** error **label** and **operational definition**; operative **phase** (pre-/intra-/post-); intraoperative **domain** (dissection, needle passage, suture handling, knotting).
- **Contributory factors:** category (technical, cognitive, environmental, equipment, anatomic/biomechanical) and description.
- **Assessment:** tool used (**ALI**, **MARS10**, **OSATS**/checklist/GRS);
- **Outcomes:** error counts/severity/patency.

**8) Synthesis methods**

Pooling is expected to be limited; therefore, we prespecify **SWiM** reporting:

1. **Study groupings:** by **operative phase** (pre/intra/post); within intraoperative by **domain** (dissection, needle passage, suture handling, knotting).
2. **Common synthesis metric:** **direction-of-association** between error type and outcome (↓ patency / no clear association/patency).
3. **Heterogeneity exploration:** by **model** (animal/synthetic/clinical), vessel type (artery/vein), and **time point**.
4. **Presentation:** structured **phase/domain tables**

**9) Amendments & deviations**

This protocol was developed a priori but **not registered**. Any changes made after protocol finalisation are documented here and in **Supplement** with date, reason, and potential impact on findings.

**10) Patient/public involvement**

None.

**11) Ethics and dissemination**

No ethics approval required (secondary analysis of published data). Results will be disseminated via a peer-reviewed journal and conference presentations. **Data and materials** will be shared in **Supplement**.

**12) Roles (CRediT taxonomy)**

- **Conceptualization/Methodology:** [VE], [VV], [GCD]
- **Search strategy:** [VE],[VV] with librarian input
- **Screening/Selection:** [EM], [OG]
- **Data extraction:** [EM], [VE]
- **Analysis/Synthesis:** [VE],[EM], [OG]
- **Writing—original draft:** [VE], [EM]
- **Writing—review & editing:** [VE],[EM],[OG],[SA],[AS],[CP],[TC],[HAC],[GCD],[CD],[TRM],[DV],[VV]
- **Supervision:** [VV]

**13) Appendices referenced**

- **Supplementary Appendix 1: File S1- Study protocol.**
- **Supplementary Appendix 2: File S2- Full search strategies & database summary.**
- **Supplementary Appendix 3: Risk of Bias assessment.**
- **Supplementary Appendix 4: Detailed, per-study characteristics**
- **Supplementary Appendix 5: Figure S1 PRISMA 2020 flow diagram**
- **Supplementary Appendix 6: PRISMA 2020 - 27 item checklist**
