# Supplementary material for: Microvascular errors of technique: a systematic review
Source: Acta Neurochir (Wien). 2026 Mar 8;168(1):65. doi: 10.1007/s00701-026-06810-w (PMC12971797; doi:10.1007/s00701-026-06810-w)
Supplement: Supplementary file 2 — Supplementary Material 2 (DOCX 15.5 KB) [file 701_2026_6810_MOESM2_ESM.docx]

Annex 2: Full search strategies & database summary;

1. Medline: (exp Microsurgery / OR (microsurg* OR microneurosurg* OR micro-neurosurg* OR supermicrosurg* OR (micro* ADJ3 surg*) OR microanastomo* OR micro*-anastomo* OR ((microvascul* OR micro-vascul*) ADJ3 surg*)).ab,ti,kf.) AND (Medical Errors / OR (error* OR mistake*).ab,ti,kf.) AND english.la. NOT (* Mohs Surgery/ OR (mohs*).ti.)
2. Embase: (microsurgery/exp OR (microsurg* OR microneurosurg* OR micro-neurosurg* OR supermicrosurg* OR (micro* NEAR/3 surg*) OR microanastomo* OR micro*-anastomo* OR ((microvascul* OR micro-vascul*) NEAR/3 surg*)):Ab,ti,kw) AND ('medical error'/de OR 'surgical error'/de OR (error* OR mistake*):ab,ti,kw) AND [english]/lim NOT ('Mohs micrographic surgery'/mj OR (mohs*):ti)
3. Web of Science: TS=(microsurg* OR microneurosurg* OR micro-neurosurg* OR supermicrosurg* OR (micro* NEAR/2 surg*) OR microanastomo* OR micro*-anastomo* OR ((microvascul* OR micro-vascul*) NEAR/2 surg*)) AND TS=(error* OR mistake*) NOT TI=(mohs*)
