# Supplementary figures and images for: Microvascular errors of technique: a systematic review
Source: Acta Neurochir (Wien). 2026 Mar 8;168(1):65. doi: 10.1007/s00701-026-06810-w (PMC12971797; doi:10.1007/s00701-026-06810-w)

## Microvascular errors of technique: A Systematic Review

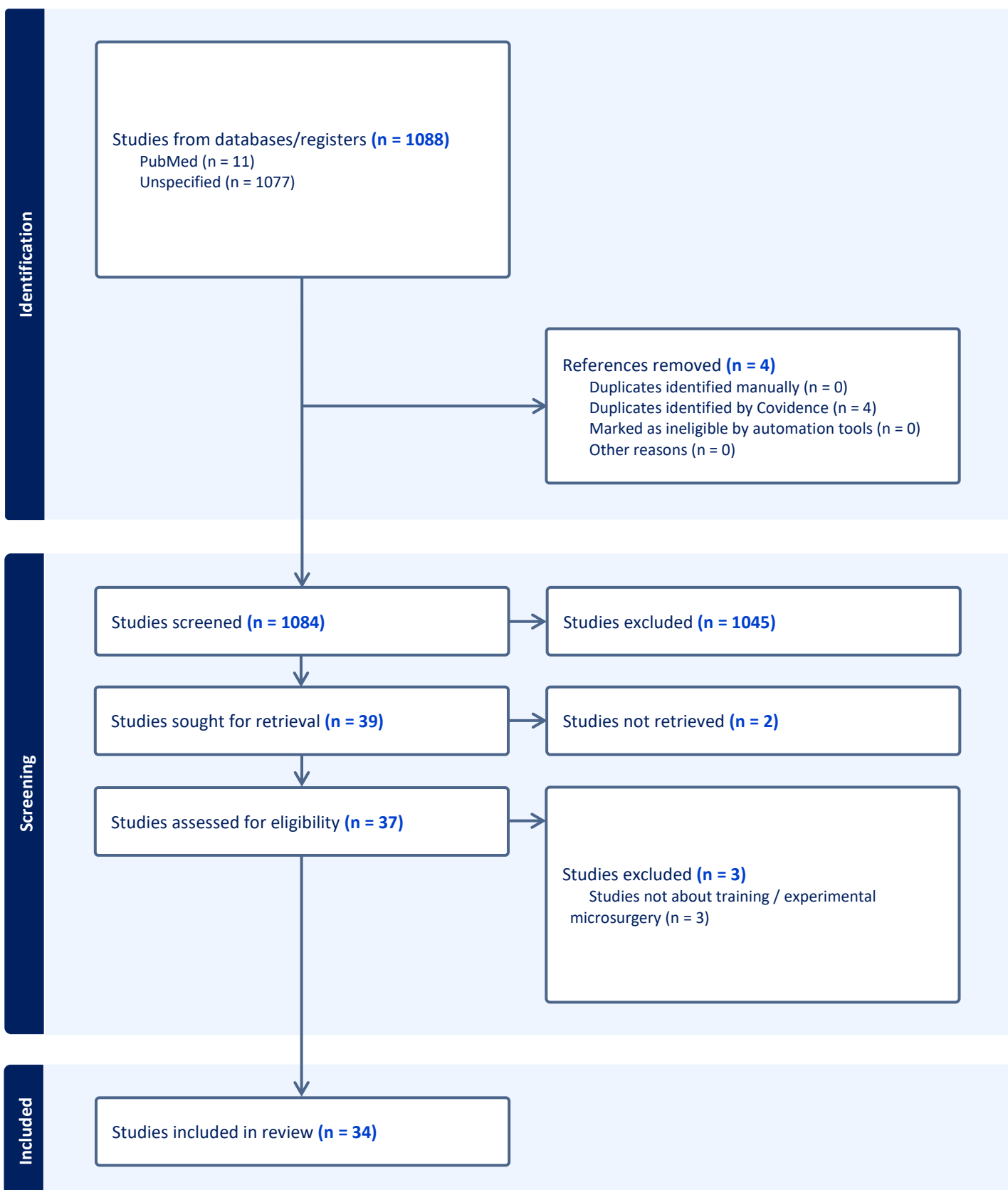

Supplement: Supplementary file 5 — Supplementary Material 5 (PDF 171 KB) [file 701_2026_6810_MOESM5_ESM.pdf]
